# Comparison of post-operative bleeding incidence in laser hemorrhoidoplasty with and without hemorrhoidal artery ligation: a double-blinded randomized controlled trial

**DOI:** 10.1186/s12893-022-01594-z

**Published:** 2022-04-21

**Authors:** Shu Yu Lim, Retnagowri Rajandram, April Camilla Roslani

**Affiliations:** Department of Surgery, Faculty of Medicine, Universiti Malaya, 50603 Kuala Lumpur, Malaysia

**Keywords:** Colorectal surgery, Hemorrhoidal artery ligation, Hemorrhoids, Hemorrhoidectomy, Laser hemorrhoidoplasty

## Abstract

**Introduction:**

The effectiveness of hemorrhoidal artery ligation supplementation in reducing the incidence of post laser hemorrhoidoplasty bleeding has not been investigated.

**Methods:**

This was a double-blind, randomized controlled trial comparing post-operative bleeding incidence in patients undergoing laser hemorrhoidoplasty (LHP) only versus LHP with hemorrhoidal artery ligation (HAL). Outcome measures included post-operative bleeding and its severity (i.e. verbal rating scale and Clavien-Dindo classification), presence of perianal swelling and pain score (visual analog score) at 1-day, 1-week and 6-weeks post-operatively. Statistical tests were performed and a value of P < 0.05 was considered significant.

**Results:**

Seventy-six patients were randomized. There was no difference in median operating time. The bleeding incidence was highest at 1-week post-operatively (17.1%), and decreased to 1.3% at 6-weeks. There was no significant difference in bleeding incidence between both groups at any of the measured timepoints (P > 0.05). Severity of bleeding and incidence of post-operative perianal swelling were similar in both groups (P > 0.05). There was no difference in median pain scores.

**Conclusion:**

Supplementation of HAL to LHP does not reduce the post-operative bleeding incidence. LHP is sufficient as a stand-alone procedure for treating haemorrhoids.

*Trial registration*: National Registration Number is NMRR-15-1112-24065 (IIR). The trial start date was 1st January 2015 with the ClinicalTrials.gov identifier and registration number as NCT04667169.

## Background

Symptomatic haemorrhoids have an estimated prevalence of 38.9% in adults [1]. These reported approximations do not include haemorrhoid sufferers who evade medical help and rely on over-the-counter medications [2, 3]. Non-excisional hemorrhoidal procedures have been gaining popularity as a treatment for these symptomatic haemorrhoids as these procedures are significantly less painful as minimally invasive procedure [4–6]. Furthermore, post-operative complications and recurrence rates are comparatively low for non-excisional procedures [7].

Lasers such as Nd:YAG have been used to perform excisional hemorrhoidectomies [8]. On the other hand, Laser Hemorrhoidoplasty (LHP) utilizes diode lasers, in a non-excisional manner, and is associated with reduced post-operative pain. It is particularly useful for grade 1–2 haemorrhoids, where prolapse is less significant [6, 9]. However, a relatively common complication is post-operative bleeding, with Clavien-Dindo Class III bleeding in the region of 1–5% [10]. Theoretically, ligating the hemorrhoidal arteries in a manner similar to the Doppler-guided hemorrhoidal artery ligation (HAL) procedure, could reduce the incidence of post-operative bleeding. Moreover, HAL can be safely performed without Doppler guidance to save time [11]; as the number and location of branches of the superior hemorrhoidal arteries are relatively constant [12].

The incidence of bleeding and recurrence of prolapse haemorrhoids for LHP has not been well reported [13] and can be high as 13% [14, 15]. While the incidence of postoperative bleeding for HAL without doppler guidance has lately been reported to be as low as 1% [16] range up to 70% [17, 18]. Theoretically, using HAL as an adjunct to LHP could reduce the incidence of bleeding, but this has not been formally assessed. We aimed to determine the effectiveness of HAL supplementation to LHP in reducing the incidence of postoperative bleeding. Post-operative pain, presence of perianal swelling and operating time associated with the two techniques were also evaluated.

## Methods

### Study design

**Fig. 1:**
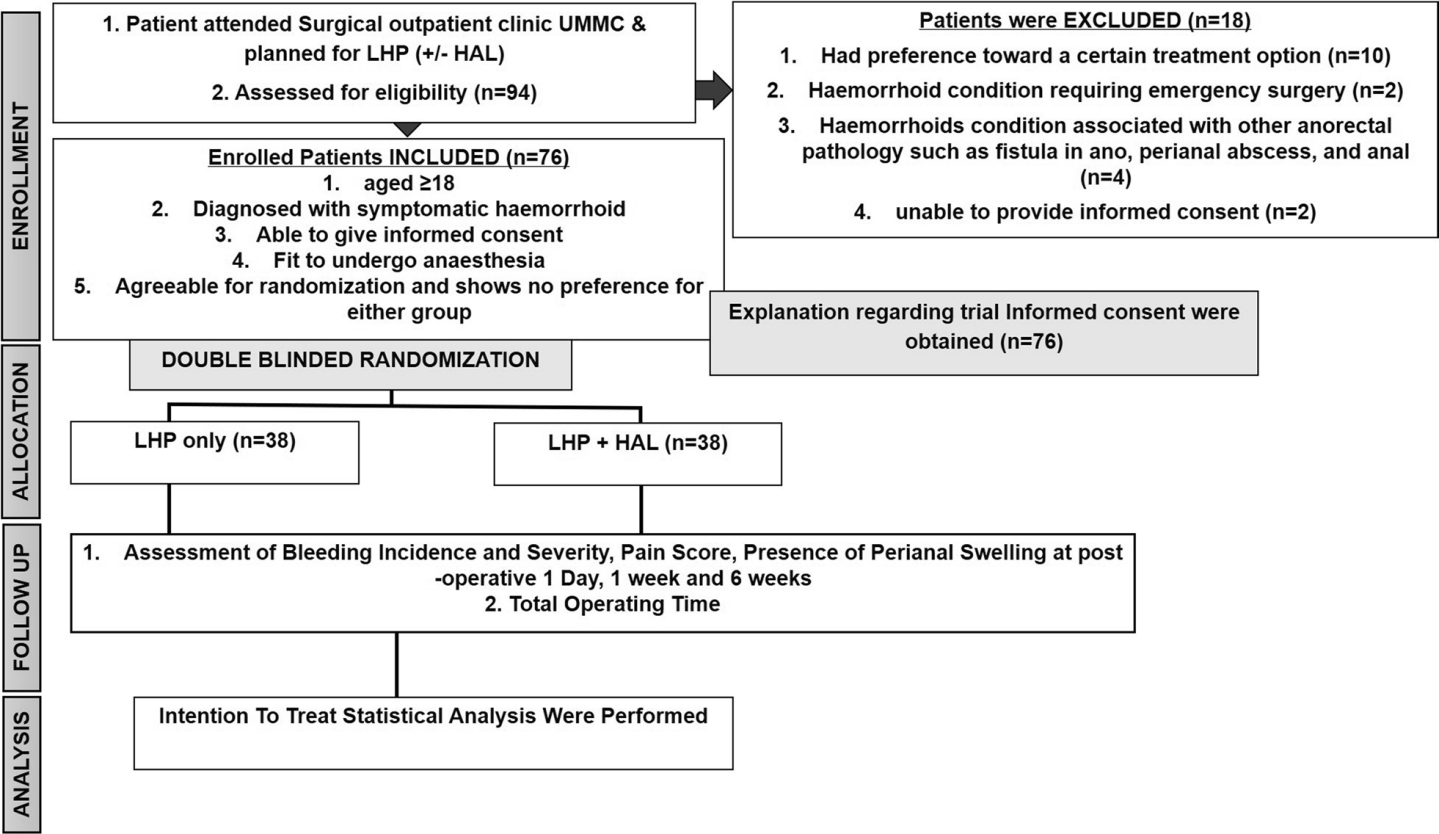
The randomized control trial consort diagram

An interventional study is designed as a double-blinded randomized controlled trial to look into the post-operative bleeding incidence of patients undergoing LHP only versus LHP plus HAL at University of Malaya Medical Centre (UMMC) (Fig. 1). The study commenced in November 2016 and completed recruitment in October 2018.

### Study population

All patients ≥ 18 years diagnosed with symptomatic haemorrhoids and failed medical therapy, fit to undergo anaesthesia and able and willing to provide written informed consent to be randomised without preference to either group LHP or LHP plus HAL were eligible to be included in this study. Patients with haemorrhoidal conditions requiring emergency surgery, or associated with other anorectal pathology such as fistula, perianal abscess, and anal fissure, were not excluded from this trial.

### Study intervention

LHP access for all patients was through stab incisions at the ano-cutaneous junction, followed by submucosal tunneling to the pedicle of the haemorrhoids with artery forceps. The laser catheter was introduced submucosally towards the pedicle guided by a visible beam to ascertain the exact location of the laser fiber. Ceralas D 50 Evolve Laser (Biolitec AG, Jena, Germany), a 980 nm diode laser, was used. Pulsed laser energy, each lasting 3 s, was subsequently delivered at 5 mm intervals, while gradually withdrawing the laser catheter. Patients in the HAL supplementation group underwent suture-ligation (especially for dearterialization) of each identified pedicle without Doppler guidance. Here, 2–0 coated Vicryl Plus Violet 70 cm CT-2 (needle used) sutures (Ethicon) were employed. In all cases no more than one suture was required even if more than one hemorrhoidal column needed suture ligation. Post-operative analgesia was standardized to oral paracetamol 1gm six hourly, and oral celecoxib 200 mg 12-hourly, for 5 days. Syrup Lactulose 15 mL 12-hourly for 1 week to prevent constipation. Subjects were discharged at the discretion of the surgeon based on discharge criteria for post-operative bleeding (VRS) and its severity (Clavien-Dindo Classification), presence of perianal swelling and pain score (VAS).

Patients had general anaesthesia (n = 16) or regional anaesthesia (n = 60) and were blinded to the type of operative procedure. Of the patients on general anaesthesia, 10 were in the LHP group while 6 were LHP with HAL group. It was anticipated that method of anaethesia would not affect the pain score at 24 h post-operation, as the half-lives of either method would have been exceeded. Prophylactic antibiotics, comprising intravenous cefoperazone 2gm and intravenous metronidazole 500 mg, were given at induction. Intravenous Ciprofloxacin 400 mg was used if patients were allergic to the aforementioned antibiotics. Once the patients were anaesthetised, they were positioned in lithotomy. Randomization of the procedures was performed using sealed envelopes. Procedures were performed through an anoscope inserted into the anus. Two drugs used for Clavien-Dindo Grade II/III type complications were tranexamic acid, and Daflon, which is a micronized flavonoid; operative interventions for Grade III complications could include hemostatic suturing or topical hemostats.

### Study blinding

A single surgeon with prior experience in over 100 LHP surgeries and over 70 HAL without doppler previously [19] performed all the cases in this study. Patients and independent observers (dressing clinic staff nurse) who assessed the presence and severity of bleeding) were blinded to the intervention. All patients were assessed in the clinic by the independent observer at 1-day, 1-week and 6-weeks post-operatively.

### Outcome measures

**Fig. 2:**
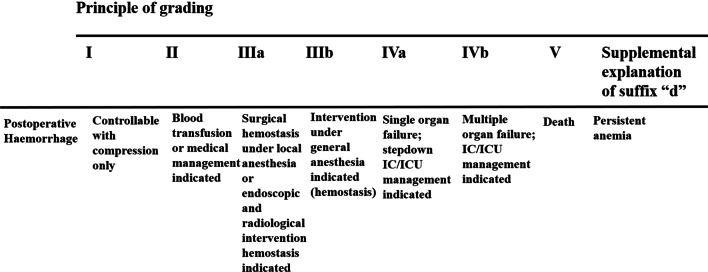
Grading of the extended Clavien-Dindo classification for post-operative haemorrhage [7]

The primary outcome measure was overall postoperative bleeding, with sub-analysis at 1-day, 1-week and 6-weeks postoperatively. Severity of bleeding is measured using the verbal rating scale (VRS) where no bleeding (= 0), mild bleeding (= 1) is defined as minimal trickling or spotting of blood, moderate bleeding (= 2) is where the bleeding is manageable with medical treatment, not needing a second procedure to stop the bleeding. Severe bleeding (= 3) is defined as significant bleeding causing a drop in hemoglobin level, needing a second operation or procedure to arrest the bleeding. While the extent of intervention for post-operative; haemorrhage was categorized using the Clavien-Dindo Classification [20, 21] as detailed in Fig. 2 [22]. Additionally, operating time was logged, presence of perianal swelling using a binary decision without severity classification was documented. Moreover, pain was recorded employing the 10-point visual analog scale (VAS) [23]. These assessments were observed and conducted by a single trained nurse blinded to the randomisation.

### Randomisation

Randomization using sealed envelopes in blocks of four was utilized to assign subjects into the two groups (LHP only and LHP with HAL).

### Sample size estimations

To show the effectiveness of supplementation of HAL to LHP in reducing the post-operative bleeding incidence by 20% [24], we needed to study 35 experimental subjects and 35 control subjects to be able to reject the null hypothesis that the failure rate for experimental and control subjects are equal with probability (power) 0.8. The Type I error probability associated with this test of this null hypothesis is 0.05. When a 10% dropout rate was factored in, we needed 38 subjects in each arm. The uncorrected chi-squared statistic was employed based on an intention-to-treat model.

### Statistical analysis

Categorical descriptive data were expressed as number and percentage and continuous descriptive data were expressed as mean ± standard deviation (SD) unless otherwise stated. Categorical data were analyzed using Chi-square analysis. All continuous variables were tested for normal distribution by the Shapiro–Wilk test. Normally distributed variables were analyzed using Student’s T-test. Continuous variables which were not normally distributed were analyzed using the non-parametric Mann–Whitney U-test. A value of P < 0.05 was considered statistically significant using SPSS version 22.0.

### Ethical considerations

The protocol of the study was registered to Medical Ethics Committee, UMMC, Medical Research and Ethics Committee and ethical approval was obtained from National Medical Research Register (NMRR). NMRR approval was obtained prior to the commencement of the study with the number NMRR-15-1112-24065 (IIR). The trial was also registered with ClinicalTrials.gov [NCT04667169]. There were no withdrawals or drop-outs from the study.

## Results

### Characteristics of study participants

A total of 76 patients were recruited for this study. Mean age of the study population was 49.8 ± 13.8. There was an almost equal number of males (44.7%) and females (55.3%). Ethnic distribution recorded 51.3% Chinese, 43.4% Malay, 3.94% Indian and 1.3% were foreigners. In regards to the grade of haemorrhoids, most were grade II (52.6%) and III (46.1%). The characteristics of patients in both groups were comparable (Table 1). Both groups did not differ statistically in terms of distribution of age, gender, ethnicity and the grades of the haemorrhoids (P > 0.05). There was no incidence of primary haemorrhage during surgery. None of the patients was on anticoagulants, nor did they have altered coagulation in their pre-operative assessment. Subjects were discharged on the same day or 1 day after surgery.

**Table 1:** Socio-demographic characteristic and supplementation of haemorrhoidal artery ligation in patients undergoing LHP

Socio-demographic	LHP n = 38 (%)	LHP + HAL n = 38 (%)	P value
Gender			
Male, n = 34	20 (52.6)	14 (36.8)	0.166^a^
Female, n = 42	18 (47.4)	24 (63.2)	
Age in years, mean ± SD	49.95 ± 12.15	49.58 ± 15.34	0.908^b^
Ethnicity			
Malay, n = 33	17 (44.7)	16 (42.1)	0.235^a^
Chinese, n = 39	21 (55.3)	18 (47.4)	
Indian, n = 3	0 (0.0)	3 (7.9)	
Indonesian, n = 1	0 (0.0)	1 (2.6)	
Grade of haemorrhoids			
1 (n = 1)	0 (0.0)	1 (2.6)	0.569^a^
2 (n = 40)	21 (55.3)	19 (50.0)	
3 (n = 35)	17 (44.7)	18 (47.4)	

Data are presented in n (%) unless otherwise specified

*LHP* laser haemorrhoidoplasty, *HAL* haemorrhoidal artery ligation, *N/A* not applicable

^a^Chi-square test (χ^2^)

^b^Student T−test; a p value <0.05 denotes a statistically significant result

### Post-operative bleeding incidence

Overall, the LHP group had a bleeding incidence of 23.7%, while the LHP with HAL bleeding incidence was 36.8%. There was a trend in the test group of having higher rates of bleeding but this was not statistically significant. In general, the bleeding incidence was highest at 1-week post-operative period with the incidence of 17.1%. The bleeding incidence at 24-h postoperatively was 11.8%, while the incidence decreased to 1.3% at 6 weeks post-operation. There was no significant association (P > 0.05) found between the presence of bleeding incidence and supplementation of HAL in patients undergoing LHP at 1-Day, 1-Week and 6-Weeks post-operatively (Table 2).

**Table 2:** Association of bleeding incidence in supplementation of HAL in patients undergoing LHP

Bleeding incidence		Procedure		P value
		LHP n = 38 (%)	LHP + HAL n = 38 (%)	
Overall	Present	9 (23.7)	14 (36.8)	0.212^a^
	Absent	29 (76.3)	24 (63.2)	
1-day post-operatively	Present	3 (7.9)	6 (15.8)	0.480^a^
	Absent	35 (92.1)	32 (84.2)	
1-week post-operatively	Present	6 (15.8)	7 (18.4)	0.761^a^
	Absent	32 (84.2)	31 (81.6)	
6-weeks post-operatively	Present	0 (0.0)	1 (2.6)	1.000^a^
	Absent	38 (100.0)	37 (97.4)	
Severity				
Presence of mild bleeding				
1-day post-operatively (n = 9)		3 (7.9)	6 (15.8)	0.480^a^
1-week post-operatively (n = 6)		3 (7.9)	3 (7.9)	1.000^a^
6-weeks post-operatively (n = 1)		0 (0.0)	1 (2.6)	1.000^a^
Presence of moderate bleeding				
1-day post-operatively (n = 0)		0 (0.0)	0 (0.0)	N/A
1-week post-operatively (n = 6)		2 (5.3)	4 (10.5)	0.674^a^
6-weeks post-operatively (n = 0)		0 (0.0)	0 (0.0)	N/A
Presence of severe bleeding				
1-day post-operatively (n = 0)		0 (0.0)	0 (0.0)	N/A
1-week post-operatively (n = 2)		1 (2.6)	1 (2.6)	1.000^a^
6-weeks post-operatively (n = 0)		0 (0.0)	0 (0.0)	N/A

Data are presented as n (%)

*LHP* laser haemorrhoidoplasty, *HAL* haemorrhoidal artery ligation, *N/A* not applicable

^a^Chi-square test (χ^2^); a p value <0.05 denotes a statistically significant result

### Severity of postoperative bleeding

As expected, clinically significant bleeding overall was more likely to happen within the first postoperative week, as evidenced by the higher VRS score and the extended Clavien-Dindo scale (Table 3). Nevertheless, there was no statistically significant difference between the two groups with respect to severity or timing of bleeding complications. There was no difference in severity of bleeding whether it was scored by extended Clavien-Dindo classification or VRS. Bleeding on the first postoperative day was usually mild (Clavien-Dindo class I). Bleeding incidence was 7.9% in the LHP group and 15.8% in the group with HAL supplementation. Significant bleeding as defined by VRS score of 2 and 3 as well as the extended Clavien–Dindo score of II and above was 7.9% in the LHP only group and 13.2% in the LHP with HAL group, but this difference was not statistically significant (P > 0.05) (Table 3).

**Table 3:** Association between Clavien-Dindo Classification and supplementation of HAL in patients undergoing LHP postoperatively

		Procedure		P value
		LHP n = 38 (%)	LHP + HAL n = 38 (%)	
1-day post-operatively				
Clavien-Dindo Classification	Class I (n = 9)	3 (7.9)	6 (15.8)	0.480^a^
	Class II-V (n = 0)	0 (0.0)	0 (0.0)	N/A
1-week post-operatively				
Clavien-Dindo Classification	Class I (n = 6)	3 (7.9)	3 (7.9)	1.000^a^
	Class II (n = 5)	1 (2.6)	4 (10.5)	0.358^a^
	Class IIIa (n = 2)	2 (5.3)	0 (0.0)	0.493^a^
	Class IIIb (n = 1)	0 (0.0)	1 (2.6)	1.000^a^
	Class IVa-V (n = 0)	0 (0.0)	0 (0.0)	N/A
6-week post-operatively				
Clavien-Dindo Classification	Class I (n = 1)	0 (0.0)	1 (2.6)	1.000^a^
	Class II-V (n = 0)	0 (0.0)	0 (0.0)	N/A
Significant bleeding incidence (VRS 2 and 3 with Clavien-Dindo Grade II and higher)				
Present, n (%)		3 (7.9)	5 (13.2)	0.711^a^
Absent, n (%)		35 (92.1)	33 (86.8)	

Data are presented as n (%)

*LHP* laser haemorrhoidoplasty, *HAL* haemorrhoidal artery ligation, *N/A* not applicable, *VRS* verbal rating scale

^a^Chi-square test (χ^2^); a p value <0.05 denotes a statistically significant result

### Operation time

There was no difference in median operating time in LHP with or without supplementation of HAL (Table 4).

**Table 4:** Association between pain score, operative time, presence of swelling with supplementation of HAL in patients undergoing LHP postoperatively

		Procedure		P Value
		LHP n = 38	LHP + HAL n = 38	
Pain score, median (range)				
1-day post-operatively		2.00 (0.75–3.25)	2.00 (1.00–3.25)	0.515^a^
1-week post-operatively		2.00 (0.00–3.00)	2.00 (0.00–3.00)	0.768^a^
6-weeks post-operatively		0.00 (0.00–0.00)	0.00 (0.00–0.00)	0.945^a^
Operation time in minutes, median (range)		25 (20–30)	25 (20–30)	0.107^a^
Presence of swelling, n (%)				
1-day post-operatively	Present	14 (36.8)	12 (31.6)	0.629^b^
	Absent	24 (63.2)	26 (68.4)	
1-week post-operatively	Present	8 (24.1)	6 (21.1)	0.554^b^
	Absent	30 (75.9)	32 (78.9)	
6-weeks post-operatively	Present	4 (10.5)	2 (5.3)	0.674^b^
	Absent	34 (89.5)	36 (94.7)	

Data are presented as n (%) unless otherwise specified

^a^Mann–Whitney U−test

^b^Chi square test (χ^2^); a p value <0.05 denotes a statistically significant result

*LHP* laser haemorrhoidoplasty, *HAL* haemorrhoidal artery ligation

### Perianal swelling

Perianal swelling was most pronounced in both groups on the first postoperative day, with an incidence of 36.8% in the LHP group versus 31.6% in the LHP + HAL group. This was not statistically significant (P > 0.05) (Table 4).

### Pain score

The median pain score by VAS for both groups were the same i.e., 2.00 at 1-Day and 1-Week after surgery, and 0.00 at 6-Weeks after surgery. No patients in the study required additional oral or parenteral analgesia. In Table 4, there was no significant association (P > 0.05) found between pain score and supplementation of HAL in patients undergoing LHP at 1-Day, 1-Week and 6-Weeks post-operatively.

## Discussion

Postoperative bleeding is a common and troublesome complication after hemorrhoidal surgery; thus, it is important to incorporate interventions with proven benefit in this regard. It was anticipated that the addition of HAL to LHP would be beneficial by lowering the incidence of postoperative bleeding, and thus reducing patient morbidity while increasing patient satisfaction.

Interestingly, our results did not show an advantage for the addition of HAL to LHP in decreasing the incidence of postoperative bleeding; in fact, there was a trend in the opposite direction, although not statistically significant. Although in principle HAL may seemingly reduce postoperative bleeding rates by dearterialization (i.e., by suture ligation) of the hemorrhoidal arteries [11, 12], this did not translate into clinical outcomes. This may be because LHP already coagulates the same vasculature by virtue of the laser ablation [25]. Furthermore, these are consistent with some developmental theories of haemorrhoids [26] and with the postulations that all dearterialization techniques have the advantage of preserving the anatomy and physiology of the anal canal [27]. In addition, the application of suture ligation creates additional sites for bleeding at the point of needle entry.

Most post-operative bleeding occurred within the first week, was mild and resolved spontaneously. The timing of bleeding after haemorrhoid surgery can be generally divided into immediate and delayed. Immediate bleeding is described as bleeding occurring within the first 48 h of a procedure. The mechanism behind immediate bleeding is probably related to the loss of control of the vascular pedicle. Delayed bleeding, on the other hand, is defined as per rectal bleeding up to 2 weeks following the procedure which is more likely due local trauma or infection [5, 28]. Additionally, delayed bleeding may be influenced by postoperative analgesia especially when NSAIDS are being prescribed which can increase the incidence of bleeding [29].

This study revealed that postoperative bleeding rarely occurred in the post-operative period of 6 weeks. This phenomenon may be explained by the mechanism of action of the laser ablation itself. LHP results in gradual fibrosis of the hemorrhoidal tissue over a period of 4–6 weeks. Hence, most of the post-operative bleeding, if any, would naturally occur prior to this period. Fortunately, most per rectal bleeding following haemorrhoid surgery will resolve spontaneously. For those that do not, treatment would depend on two factors; the location of the bleeding and the degree of blood loss. External measures such as tamponade or compression with gauze or suture ligation at the bedside are usually successful in arresting the bleeding. Injection of local anaesthetic with adrenaline can also be performed at the bedside with a good success rate although such bedside procedures may be uncomfortable for the patient. Up to 15–33% of patients with bleeding after haemorrhoid surgery would be required for a re-operation [30, 31].

It is known that stapled haemorrhoidopexy has a higher re-bleeding rate compared to excisional haemorrhoidectomy with rates over 30% [32]. Surprisingly, most of the re-bleeding cases will not have an identifiable source of bleeding by the time they are examined in the operating room and more often than not, a sponge or tamponade will just be placed in the anal canal. However, these significant bleeding episodes may even be recurrent and cause distress to both the patient and the surgeon [31] Here only a small percentage (2.63%) of re-bleeding patients needed a second procedure or operation to arrest the bleeding; i.e., two cases, one per group were re-admitted. These results are comparable to studies on post-operative bleeding in LHP in other centres [1, 5, 11, 33].

Despite the addition of HAL to the LHP, there was no significant difference in the operation time in both groups. This may be explained by the fact that the study was conducted in a colorectal centre, and the surgeons were familiar with both techniques. Hypothetically, if the procedure is performed by inexperienced surgeons, additional procedures on top of LHP may significantly prolong the operating time [34]. Furthermore, an additional (but unnecessary) procedure may pose an increased risk of complications for the patient. Although most trials involving HAL were associated with a low re-bleeding rate, one study has been shown to have a postoperative bleeding rate of as high as 70% [18].

Perianal swelling was also completely resolved by the end of 6 weeks of surgery. In regards to the pain score (VAS), there was no clinical or statistically significant difference in between both groups since both LHP and HAL are minimally invasive procedures and non-excisional in nature [4–6]. In fact, most patients only experienced mild pain (median pain score = “2.00”) which were easily controlled with oral paracetamol and celecoxib.

### Study limitations

There were no prior studies looking into the bleeding rate for LHP with HAL, therefore the sample size for this study was calculated based on a surrogate bleeding rate from HAL from another study model. This study design focused on a short-term follow-up at a single-centre only. Larger randomized trials are awaited to demonstrate the bleeding incidence, long-term outcomes (up to 2 years) and efficacy of combined LHP procedures is warranted.

## Conclusion

Supplementation of HAL to LHP does not decrease the post-operative bleeding incidence.

## Data Availability

Not applicable.

## Abbreviations

HAL: Hemorrhoidal artery ligation
LHP: Laser hemorrhoidoplasty
NdYAG: Neodymium-doped yttrium aluminium garnet
NMRR: National Medical Research Register
SD: Standard deviation
UMMC: University of Malaya Medical Centre
VRS: Verbal rating scale
VAS: Visual analog score
